# The Impact of Halloysite on the Thermo-Mechanical Properties of Polymer Composites

**DOI:** 10.3390/molecules22050838

**Published:** 2017-05-20

**Authors:** Tayser Sumer Gaaz, Abu Bakar Sulong, Abdul Amir H. Kadhum, Ahmed A. Al-Amiery, Mohamed H. Nassir, Ahed Hameed Jaaz

**Affiliations:** 1 Department of Mechanical & Materials Engineering, Faculty of Engineering & Built Environment, Universiti Kebangsaan Malaysia, Bangi, Selangor 43600, Malaysia; 2 Department of Machinery Equipment Engineering Techniques, Technical College Al-Musaib, Al-Furat Al-Awsat Technical University, Al-Musaib, Babil 51009, Iraq; 3 Department of Chemical & Process Engineering, Faculty of Engineering & Built Environment, Universiti Kebangsaan Malaysia, Bangi, Selangor 43600, Malaysia; amir@eng.ukm.my (A.A.H.K.); dr.ahmed1975@gmail.com (A.A.A.-A.); 4 Program of Chemical Engineering, Taylor′s University-Lakeside Campus, Subang Jaya, Selangor 47500, Malaysia; mhnassir1949@gmail.com; 5 Solar Energy Research Institute (SERI), Universiti Kebangsaan Malaysia, Bangi, Selangor 43600, Malaysia; eng_tay83@yahoo.com

**Keywords:** polymer-matrix composite, nanostructure, mechanical properties, physical properties, mechanical testing, thermal analysis

## Abstract

Nanotubular clay minerals, composed of aluminosilicate naturally structured in layers known as halloysite nanotubes (HNTs), have a significant reinforcing impact on polymer matrixes. HNTs have broad applications in biomedical applications, the medicine sector, implant alloys with corrosion protection and manipulated transportation of medicines. In polymer engineering, different research studies utilize HNTs that exhibit a beneficial enhancement in the properties of polymer-based nanocomposites. The dispersion of HNTs is improved as a result of pre-treating HNTs with acids. The HNTs’ percentage additive up to 7% shows the highest improvement of tensile strength. The degradation of the polymer can be also significantly improved by doping a low percentage of HNTs. Both the mechanical and thermal properties of polymers were remarkably improved when mixed with HNTs. The effects of HNTs on the mechanical and thermal properties of polymers, such as ultimate strength, elastic modulus, impact strength and thermal stability, are emphasized in this study.

## 1. Introduction

**Figure 1:**
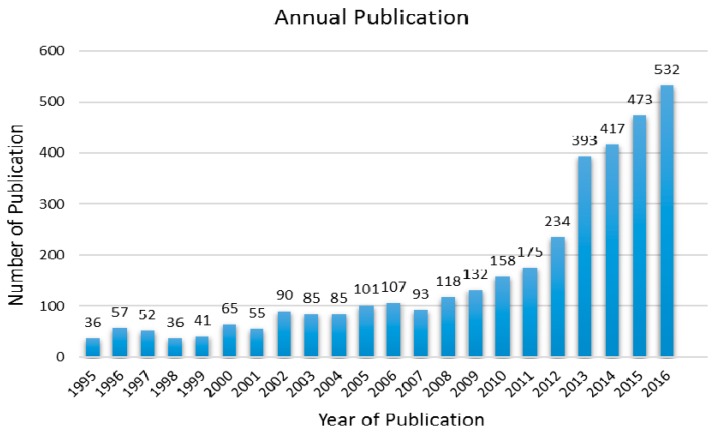
Annual number of scientific research publications on halloysite in the past two decades (using the SciFinder Scholar search system to obtain the above data, as of December 2016).

Halloysite Nanotubes (HNTs) are a naturally-occurring aluminosilicate (Al_2_Si_2_O_5_(OH)_4_·2H_2_O) with a predominantly hollow tubular structure mined from natural deposits [1,2,3,4,5,6,7,8]. Du et al. described HNTs as a clay with a hollow nanocylindrical structure excavated naturally from earth deposit resources in many areas, including China, Brazil and France [4,9,10,11]. HNTs are naturally processed through weathering of minerals in Earth′s environment and are exploited to a great extent as sorbents, nanocontainers and composite polymer matrices reinforced with nano-scale fillers [12]. HNTs are characterized further with distinct mineral compositions and structures that could be used to supplement polymers and have unique reinforcing effects on various polymers [11,13,14,15]. With the unique crystal structure and the surface ability [16], HNTs are characterized by a hydroxyl with a lower level of density that permits smooth diffusion in a polymer matrix compared to other nanoclays [17,18]. For these reasons, halloysites are considered as an excellent candidate for polymer composites and have become recently of great research interest [15]. As an additive, HNTs are used for attaining advanced mechanical and/or thermal features [19]. The crystalline structure marks the singularity of the HNTs, as no exfoliation is required, and the intercalation of the HNTs by polymers and additives is difficult to achieve due to the small basal spacing of the crystal planes. Furthermore, the polarity of the tubules surface shows that the HNTs are suitable to secure generous dispersal in the polymer matrices [20]. Ismail et al. [21,22] noted that HNT nanoclays can be used as nanocomposites, nanocontainers and adsorbents [23,24]. In relation to the halloysite as a research topic, Figure 1 shows a publication survey for the last two decades taken from the SciFinder Scholar search. The increasing interest is readily clear from the growing number of publications, which amounts to about a constant rate of 13% during the last two years.

The ability of HNTs to absorb some hazardous materials such as hexavalent chromium (Cr^+6^), which is widely used in industry, can be considered as one of its most important applications. The utilization of all clay minerals in nanocontainers, nanocomposites and adsorbents render HNTs’ usefulness as a vital component in advanced materials nowadays. Elisa et al. [18] immobilized hematin onto HNTs, which were evaluated as biomimetic catalysts for the environmentally-friendly polymerization of aniline in aqueous acidic media. Additionally, HNTs are portrayed as potential adsorbents for organic pollutants and metal ions due to the large focused surface domain, hollow and layered structure [25].

HNTs are considered as a more favorable option than kaolin and wollastonite. By choosing HNTs with a high aspect ratio, high potential composites for particular applications and target properties can be achieved. Thus, HNTs appear to be an alternative of interest because of lower cost in the fabrication of high-performance materials as polymers and a cost-effective option for particular uses compared with using other costly nanofillers, like CNTs [26]. In neutral situations, the surface of HNTs becomes negatively charged [27,28]. Xie et al. [29] showed that HNTs can absorb more cationic dyes and react with dispersions in the water due to their hydrophilic surface. At the beginning of contact, fast adsorption on the HNT surface may result from the vast availability of active sites [30].

Researchers claim that the natural accumulation of aluminosilicate nanotubes and the material′s implementation within the context of rubber industries as nanocomposites are as yet predominately not fully explored [31]. HNTs have two layers with mostly a hollow slender cylindrical structure in the submicron ambit, which is smaller than one millionth of a meter, and its chemical composition is identical to kaolin [31]. Moreover, scientists showed that in the unmodified nanotube surface, the resulting nanocomposites overwhelmingly proffer poor HNTs dispersion in polar matrices combined with a restricted improvement of the eventual characteristics [23].

The field of nanotechnologies has many applications in advanced materials and nanocomposites. In particular, nanotubes represent the most effective research in terms of nanotechnology [24]. In addition to the unique characteristics of HNTs and despite the similarity of the chemical character of HNTs and kaolin, HNTs are still preferable to be used in applications like gas separation. Based on these desired characteristics, the existing water molecules in a monolayer that separates the unit layers in HNTs and the larger inner cavity, HNTs can accommodate molecule sizes ranging from light gas to protein molecules [32]. HNT deposits have been discovered in different parts in a range of particle and hydration conditions [33]. Tang et al. [34] reported that the capability of using salts and organic compounds to intercalate or exfoliate halloysite is obvious. From the structure, treatment of HNTs resulted in leaching of the diaspore-like sheets [35]. Thus, more focus is given to HNTs because of the unique structural characteristics and the chemical stability [36]. HNTs having stable properties are able to resist against organic solvents and exhibit reusability advantages [37]. Considering that HNTs possess reactive hydroxyl groups on the tube surface, they can be changed in terms of certain organic properties for increased adsorption selectivity of metal ions [38]. The main disadvantages of HNTs are the properties of being inorganic and negative biocompatibility, thereby leading to low enzyme loading and a weak link between enzyme and nanotube [39]. Tubular halloysite was utilized as an adsorbent, nanotemplate and catalyst enhancer [40]. Halloysites are capable of entrapping molecules with certain sizes and have been utilized as a good nanocage for active molecules because of the space of the nanotubes [41]. Furthermore, new research shows that HNTs are biocompatible and can be exploited as biomaterials [42]. HNTs were also treated with biopolymers, and the morphology was studied, which has shown that the sandwich-like structure of the nanocomposites is strongly dependent on the nature of the polymer [43,44]. More importantly, HNTs has very important applications in the medical fields in particular. Creating functional biopolymer film was performed recently [45]. The film was prepared by adding a pectin matrix to HNTs in an essential peppermint oil. The process requires fictionalizing HNT surfaces with cucurbit uril molecules.

In the combustion process, the mechanism of action of clay nanotubes resulted in the surface accumulation of the condensed time and the accumulation of homogeneous inorganic left-over elements that can negatively affect heat and mass transport [46]. A preferable dispersal dynamics was exhibited within the structure matrix of the host polymer ‘the epoxy’ by utilizing the nanoplatelets as a result of unfolding and the intercalated halloysite compared with using the unconditioned halloysite according to a study by Youhong et al. [47]. Jiang et al. [48] noted that HNTs have common open-ended pores and a larger pore radius than that of zeolite. Prashantha et al. [49] deduced that HNTs are a class of a matchless eco-friendly, sustainable material of auspicious reinforcement capability for thermoplastics.

The dispersion of HNTs is very important in nanocomposite technology. Attempts have been mounting to achieve better dispersion hoping to form a new generation of environmentally-friendly nanocomposites by selecting two pectins of different degrees of methyl esterification and nanoclays like halloysite and laponite [50]. HNTs’ characterization as an additive and as nanocomposite has been extensively studied using several tools. In one of these studies, it has shown that small quantities of HNTs could generate a better compact structure than a big HNT quantity [44].

In this review, HNTs’ properties that are the most effective on the mechanical and thermal properties of HNTs are reviewed. The review focuses on the highest level of the published papers and, occasionally, considers lower levels only under severe conditions. Out of many characterization techniques, FTIR, SEM and TEM for structural analysis, tensile strength and elongation at break for mechanical properties and TGA and DMA for thermal properties are included in this review.

## 2. Structure of HNTs

**Figure 2:**
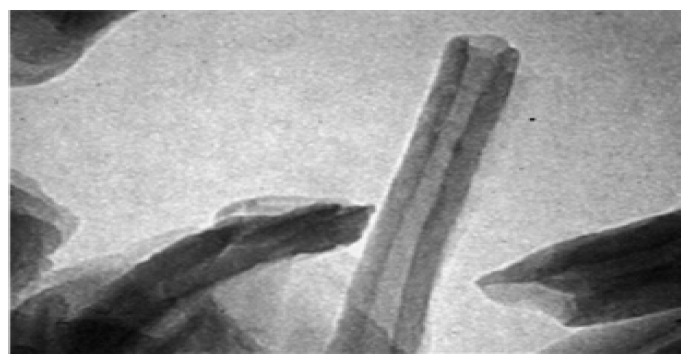
TEM micrograph of HNTs [21].

The micrographs of HNTs are elucidated in Figure 2. A predominant percentage of HNTs are of tube-shaped structures having the following measurements: length varies from 150 nm to 2 μm; the outer diameter ranges from 20 nm to 100 nm; whereas the lumen diameter ranges from 5 nm to 30 nm. Distinctive and differential morphological aspects of HNTs show uncommon charge distributions and surfaces with lower hydroxyl density, and crystals with an unequaled structure are observed [7]. More important to note is that the homogeneous dispersion of HNTs can be smoothly achieved within the Ethylene Propylene Diene Monomer (EPDM) matrix [21].

**Figure 3:**
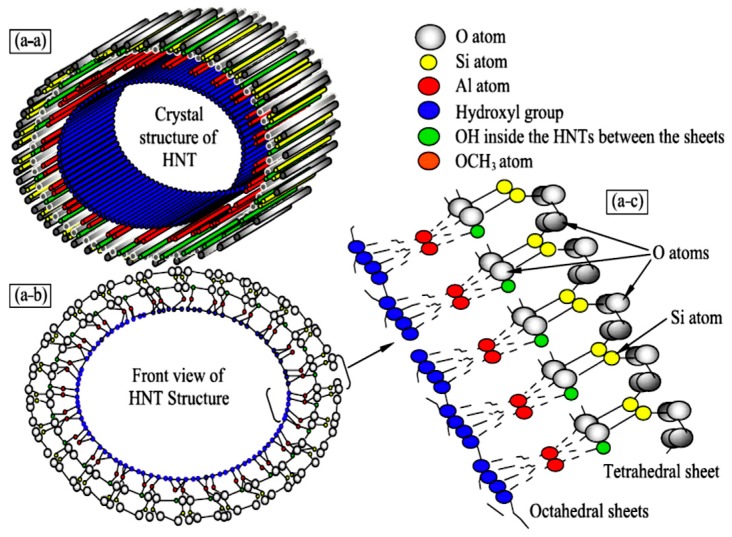
Crystalline structure of halloysite [53].

HNTs (Al_2_Si_2_O_5_(OH)_4_·2H_2_O) have two types of hydroxyl groups: the inner group, which is situated between nanotubes layers, and the second group, which is a surface hydroxyl group [51]. The color of pure HNTs is usually white; however, coloring may occur due to impurities that transform HNTs to a yellowish or even brown color. Coloring occurs depending on type and level of impurities, like Fe^3+^, Ti^4+^ and Cr^3+^ ions that impregnate the mineral [52]. Figure 3 shows the crystal structure of the HNTs, which consists of monoclinic unit cells. Three different views (a-a, a-b and a-c) for the crystalline structure of halloysite are also given. The tubular shape of halloysite contains two layers, namely, tetrahedral and octahedral [53]. The HNTs structure is still under investigation. A new structure was examined by using a computational SCC-DFTB software where the structure is described by tetrahedral and octahedral distortion, which yields a spiral nanotube [54].

## 3. Advantages of Halloysite Nanotubes

Table 1 summarizes the main properties, features and aspects of the applications of HNTs [51,52].

## 4. Applications of Halloysite Nanotubes

The addition of thin halloysite layers improves the adhesion matrix of human dermal fibroblasts. Moreover, the cellular phenotype was also maintained. Human breast cells and fibroblasts were used to test the toxicity of halloysite by incubation for 48 h. Noticeably, the test affirms the non-toxic effects of halloysite on the cells and surprisingly appears to be less harmful than daily consumed NaCl. Furthermore, even the addition of 0.1 wt % of halloysite to the cell culture did not result in cells’ death. Finally, the mechanism of cellular uptake was visualized and revealed that the nanoparticles concentrated around the cell nucleus after penetrating through the cell wall [52]. The biocompatibility of HNTs characterizes its important. Biocompatibility can be enhanced by wrapping amylase on HNTs’ surface. This biocompatibility utilization emphasizes the ability of HNTs as an excellent absorbent. Moreover, in hydrocarbon processing and catalytic conversion, HNTs can be used as a molecular hydrogen storage. In the field of environment, HNTs can be used as a diuretic drug transporter for removing hazardous species [55]. Various types of active agents that serve as drug components can be entrapped in the inner cavity of nanotubes and combined with invalid spaces of the multi-layered aluminosilicate shells of HNTs [7].

## 5. Halloysite-Polymer Nanocomposites

Table 2 summarizes the methods used to prepare and obtain halloysite-polymer composites with a brief outline of the methods and the expected and observed results [52]. Rui et al. [56] maintained that natural HNTs were used as novel materials for enhancement to immobilize enzymes. The comparison depicts that HNTs can be utilized as a convenient medium for the immobilized enzymes that can positively affect storage stability. The study concluded the possibility of using HNTs as carriers for the enzymes under study in practical applications and can also include other enzymes.

Ultrafiltration membrane was prepared based on Polyether Sulfone (PES) as the membrane material and copper ion-modified HNTs as an antibacterial agent. The hybrid membrane has enhanced mechanical strength due to the dispersion of chemically modified HNTs. Reduced interfacial stress resulted in additional HNTs being more harmonious with the polymer matrix. By contrast to alternative inorganic carriers, the existence of appropriate hydroxyl groups added to the tube-shaped profile of HNTs resulted in smooth dispersion for the polymer matrix [57]. At the surfaces of the HNTs, anilinium cations were absorbed because of the negative centers and as a result of the reaction of HNTs with hydrochloric acid, resulting in modification of the surfaces of the HNTs, thereby making them more hydrophobic [58]. In terms of organic rigid molecules, acidic sites of HNTs surfaces will be shielded due to the effects of benzothiazole groups [59].

HNTs were intrinsically nonselective because of the large lumen diameters; thus, the increase in selectivity can be attributed to its tortuosity [60]. At high concentrations of halloysite addition and because of the bound rubber structure, the effect may be more significant than the effects of the outer halloysite layer′s acidity, leading to reduced swelling percentages [61]. Furthermore, both the displacements at optimum loads and the entire displacements of the HNT composites were greater than the HNT composites without them [62]. The main locations of the functional group of halloysite nanotubes, like the hydroxyl group, are on the inner surface and at the end of the tube [63]. In addition, the sliding distance affects the level of increase in weight loss in direct proportion when the content of HNTs is high. The said phenomenon is attributed to the weak level of interfacial adhesion of the HNTs to the polyester as a direct consequence of the lack of an adequately appropriate resinous region to alienate the load by transferring it through to the HNTs [64].

The addition of chitosan solution to the HNTs expedites the emergence of the elevated deposition yield. The increase in deposition is due to co-deposition of chitosan and HNTs. Another reason is the build-up of composite chitosan/HNTs films. The elevation in the concentration of HNTs’ suspension concentration led to the increase in HNTs content for the deposits [65]. Moreover, the large number of Si OH groups on the external surface of the HNTs may cause a reasonable increase in the interfacial bonding sturdiness between the silanized HNTs as a modified nanofiller and the dental resin matrix as a host polymer [66]. Natural HNTs tubules are described as unique and versatile and make up the surface weathering of aluminosilicate minerals. HNTs are deposited with other clay minerals and are therefore difficult to differentiate. The distinctive features of HNTs render them advantageous in a wide array of applications; such as acting as drug containers through the agent entrapment process within the inner cavity of the tube-shaped structure using polymers not readily susceptible to fire. Other approaches may cover processes to enable features, such as controlled release rate or through the acquisition of low molecular weight elements instead of the intercalated water through the swapping process [67]. In addition, materials with controllably-varied properties were shaped and produced by enfolding HNTs into hydrogel produced via crosslinking through an electrostatic process, in which the resultant encapsulated hydrogel is composed of an internally-connected biopolymer network system [68]. Liu et al. [69] revealed that given the very small diameters of HNTs, the tubes can be deemed as structured macromolecules; therefore, the polymer chains need to absorb and align on the HNTs’ surface. HNTs operate as heterogeneous nucleating agents, with hydrogen linking on the HNTs’ surface. Interactions between the amide groups in polyamide and the hydroxyl groups could be sufficiently tough to partially prevent the movement of the polymer chains. Ample evidence has been presented by researchers to affirm the intrinsic feature of HNTs in enhancing the thermal stability of polymers and providing fire-retardant properties to the polymers [70]. Moreover, the hardening of chitosan membrane hardening when mixed with nanoclay HNTs was observed due to the improvement in the structural rigidity of the “chitosan polymers chains” [71]. The increase in rigidity is attributed to the interaction between chitosan and its neighboring HNTs. Thus, an increase in selectivity was predicted because of the silane agent being linked to the HNTs in the polymer matrix [72]. When added at high amounts, HNTs exert a moderate effect on the continuous movement of the polymer that may be still processed as neat material [73]. Incorporation of HNTs with polymers to enhance their properties has received wide interest. The integration of these nanoparticles with polymer matrix without altering the chemical structure of the host polymer advanced these topics rapidly [74]. HNT-sodium perfluorooctanoate was also recently tested by TGA to see the functionality in the paper manufacturing industry [75]. In a very recent study, the surface of HNTs was grafted by poly(*N*-isopropylacrylamide) in order to use this system for drug delivery [76,77,78].

## 6. Microstructure, Mechanical and Thermal Properties of Halloysite Nanotubes

### 6.1. Microstructure

**Figure 4:**
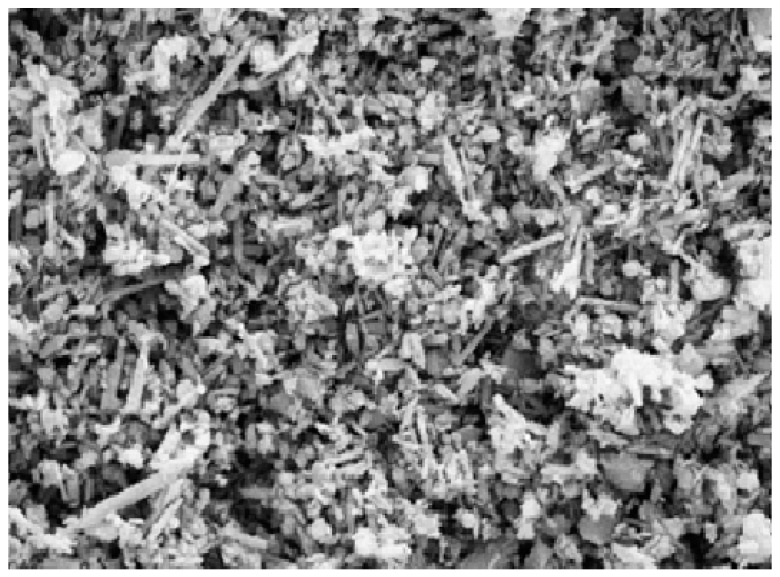
Morphological characterization of combustion residue of modified ‘HNT-based nanocomposites’ with 10-per hundred rubber (phr) loading [9].

**Figure 5:**
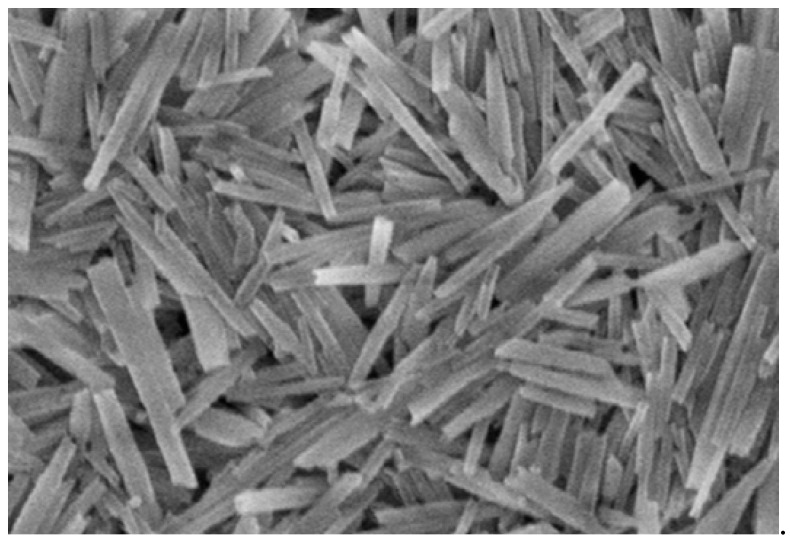
SEM results of the natural HNTs [39].

In nanocomposites with HNTs loading, the halloysite elements in the conditioned system are evenly dispersed despite the presence of some aggregates. Figure 4 illustrates the morphological characterization of combustion residue of modified ‘HNT-based nanocomposites’ with 10 per hundred rubber (phr) loading; also as indicated, keeping the tubular structure of HNTs even after the combustion process [9]. At low HNTs’ loading, the ideal morphology was observed, in which the morphology was built upon when an equal distribution of HNTs coupled with appropriate adhesion between HNTs and polymer matrix was obtained [32]. The SEM image depicts the overall morphology of HNTs shown in Figure 5. The SEM of the original HNTs′ description of the nanotube surfaces shows clear, smooth edges [39].

Agglomerates of HNTs were observable even after suspending the powder in acetone. In addition, SEM micrographs revealed that the agglomeration of halloysite was more substantial at larger filler fractions [26]. Thus, based on the morphological observations, greater enhancement of thermal stability was achieved for internally-mixed nanocomposites when compared with twin-screw compounds, whereby HNTs were preferentially based [70].

#### 6.1.1. Halloysite Nanotube and Polylactide-Based Nanocomposites

Apparently, the research study on Polylactide (PLA)-HNTs nanocomposites is either missing or has not been conducted to provide proof of the thorough impact of HNTs as a reinforcement element on PLA properties. According to [79], the attention for HNTs is relatively new to some extent. HNTs have been evaluated as potential nanofillers for PLA. Melt-compounding technology was used to produce PLA/HNT- based nanocomposites. Commercially available HNTs were added into the PLA matrix; alternatively, extrusion grade silane-treated HNTs were added. To underline performance enhancement, characterization of the resulting nanocomposites was conducted. Notably, the possibility of obtaining high-performance HNTs-PLA nanocomposites utilizing the melt-blending approach was elucidated in the first studies [79].

#### 6.1.2. Halloysite Nanotube and Poly(Butylene Succinate) Based Nanocomposites

**Figure 6:**
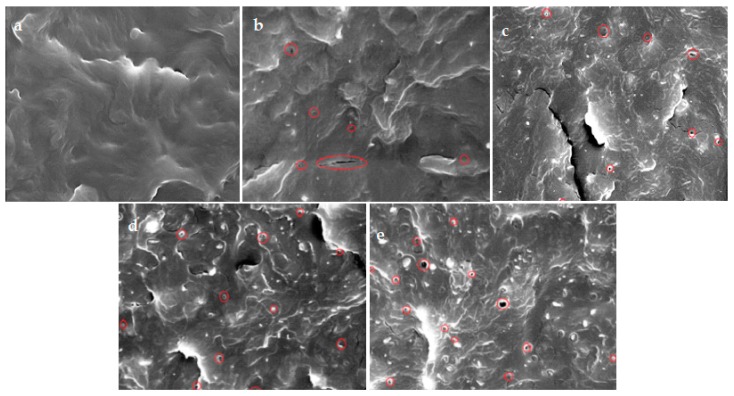
Scanning Electron Microscopy (SEM) of the nanocomposites′ fractured surface for neat PBS and PBS-HNT-based; (**a**) PBS0: neat PBS; (**b**) PBS1: PBS + 1 wt % HNT loading; (**c**) PBS3: PBS + 3 wt % HNT loading; (**d**) PBS5: PBS + 5 wt % HNT loading; and (**e**) PBS7: PBS + 7 wt % HNT loading; PBS: Poly(Butylene Succinate) [80].

The microstructure of Poly(Butylene Succinate) PBS has been investigated using Scanning Electron Microscope (SEM) micrographs. PBS samples were freeze-fractured by immersing in liquid nitrogen prior to the SEM observations (Wu et al. [80]). Fractured neat PBS SEM micrographs are shown in Figure 6a. Examining the nanofiller distribution with varying HNTs contents (1–7 wt %) using SEM micrographs shows uniform dispersion and distribution of HNTs within the PBS matrix; Figure 6b–e. Aside from some small cavities in PBS/HNT composites’ micrographs (Figure 6b–e) on the fractured PBS/HNT nanocomposites’ surface, marked by the red circles, HNTs still exhibit a uniform distribution behavior even with HNTs contents as high as 7 wt % (Figure 6e). The above observation ascertains the necessity for additional enhancements to the nanofiller HNTs’ interfacial interaction with the host polymer matrix, PB [80]. The mechanical properties were enhanced by a new technique focusing on modifying the inner surface of HNTs by introducing cycloaddition of asides and alkynes [81].

### 6.2. Fourier Transform Infrared Spectroscopy

**Figure 7:**
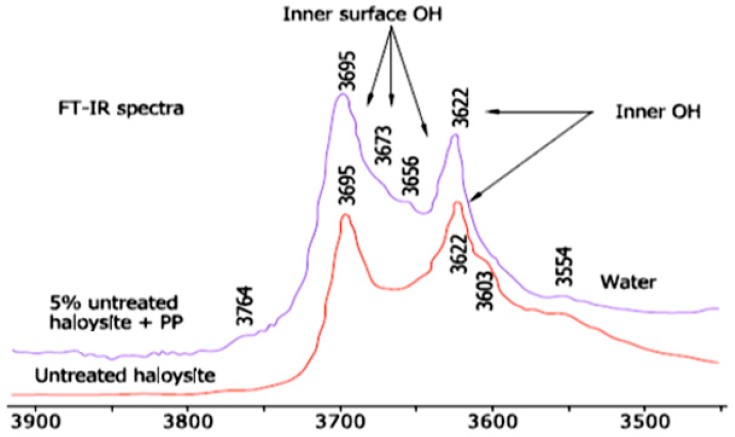
FTIR analysis of the halloysite [82].

Based on the results shown in Figure 7, the FTIR spectrum of the halloysite asserts the following notes: the band at 3695 cm^−1^ represents to the stretching vibration of the inner surface OH groups, while the band at 3622 cm^−1^ represents to the stretching band of the inner groups. The inner surface OH groups are connected to the Al-centered octahedral sheets and form hydrogen bonds with the oxygen sheet in the next double layer. Typically of halloysite, the other two inner surface OH groups that occur at approximately 3650 cm^−1^ and 3670 cm^−1^ cannot be observed [82].

### 6.3. Mechanical Properties

#### 6.3.1. Halloysite Nanotube and Polypropylene-Based Nanocomposites

Only a slight improvement of the mechanical features was observed by the regular crystallinity in the polypropylene (PP) matrix and the very small length to diameter ratio of HNTs. An increase in the mechanical features for the composites with changed halloysite compared with the unchanged composites was reported by Ulrich et al. [26]. The decrease in spherulite dimension by HNTs can result in improvement of the PP surface roughness [69]. Based on Table 3, which shows the tensile strength (σ) and Young′s modulus (*E*) of the nanocomposites containing 5 wt % HNTs, the mechanical properties of the PP were observed to be insignificantly effected when untreated HNTs were added. By contrast, treatment of HNTs with Hexadecyl-tri-methyl-ammonium-bromide (HEDA) results in an increase in PP modulus; however, this enhancement did not include the complemental increase in tensile strength. Other investigations and studies conducted on composites containing silane-modified HNTs returned similar results [82].

#### 6.3.2. Halloysite Nanotube and Epoxy-Based Nanocomposites

An increase in the elastic modulus from 130 MPa–300 MPa was observed, whereas the composite became brittle because the elongation at break decreased from 80% down to 56%. Upon the addition of 9 wt % HNTs, the composite’s tensile strength has increased from 4 MPa–10 MPa, although this value decreases with a further addition of halloysite [52]. The addition of 2% HNTs to epoxy was reported to result in a four-fold increase in impact strength. For the tensile strength, a ten-fold increase from 1.3 MPa–13 MPa was obtained. Addition of 8 wt % HNTs doubled the composite′s tensile strength from the original value of 30 MPa. Considerable enhancement in the mechanical properties of the composite materials was obtained and is attributed to the fact that Polyvinyl Alcohol (PVA) chains were subjected to orientation and crystallization caused by halloysite, thereby contributing to the aforesaid enhancement. A study by Lvov et al. [52] shows that the tensile strength increased from 9 MPa–13 MPa by the addition of 4 wt %–5 wt % HNTs.

### 6.4. Thermal Properties

#### 6.4.1. Halloysite Nanotube and Polypropylene-Based Nanocomposites

**Figure 8:**
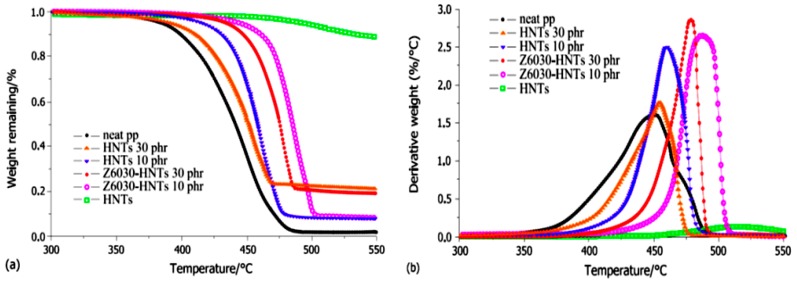
(**a**,**b**) TGA curves of neat PP, HNTs and PP/HNT nanocomposites in nitrogen [9].

**Figure 9:**
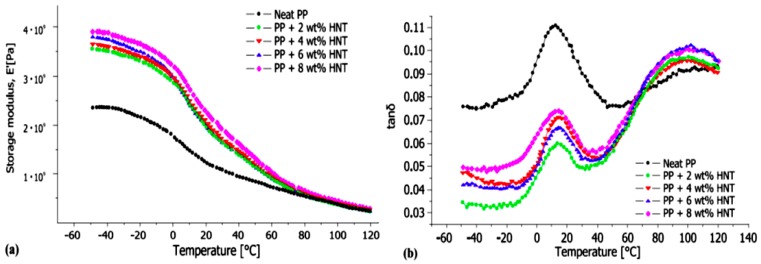
(**a**) Storage modulus (*E'*) and (**b**) Tan δ with temperature sweep as a function of nanotube content for PP/HNT nanocomposites [49].

The temperature of the nanocomposite filled with 10 phr modified HNTs as compared with neat PP is 60 °C higher at 5% weight loss in nitrogen. Thus, in addition to the entrapment operations of HNTs, the obstacle operations also have crucial functions in the thermal stability of the nanocomposites. In nitrogen, the TGA curves of PP/HNTs nanocomposites are shown in Figure 8 [9]. The dynamic storage modulus for PP and HNTs nanocomposites as a function of temperature is shown in Figure 9a. The storage modulus of PP increases with increasing HNTs content, which is due to the reinforcement effect and restrictions in the chain mobility. Figure 9b illustrates the effect of HNTs on the loss factor (tan δ) for PP nanocomposites [49].

The effectiveness of available space in halloysite tubular lumen is utilized in the entrapment of the reaction products that contribute to flame acceleration and degrading performance thermally when the polymers start to inflame, consequently shifting thermal stability degradation temperature. A 20% enhancement in thermal stability of PP can be achieved from 351 °C–425 °C by doping only 10% of halloysite to the polymer matrix. Another example is thermal stability enhancement of halloysite-pectin composites in comparison with the pure polymer. Increasing thermal degradation temperature by 5% from 238 °C down to 250 °C was achieved by increasing halloysite content from 50 wt %–80 wt %. Interestingly, an addition of a control element ‘reference’ such as platy laponite and kaolinite reveals considerably less enhancement in terms of thermal stability. Thermal stability degradation temperature of starch, which is 311 °C, shifted to 321 °C upon addition of 9 wt % halloysite, which resulted in halloysite-doped starch biocomposite material [52]. The nanocomposites’ thermal stability and fire retardant properties are heightened by the addition of HNTs [67].

The DSC results show that the presence of the HNTs induced a detrimental effect on the matrix, evidenced by a reduction in matrix crystallinity (Table 4). Moreover, by considering the significant nucleating activity of HEDA on PP, as documented by DSC (Table 4), the main benefit of HEDA appears to be compensation of the reduced matrix crystallinity. The effect can be clearly seen in the DSC results (Table 4), which indicate the presence of a significant negative effect of the urea (intercalated into the HNT) on crystallinity and, thus, the mechanical parameters of PP [82].

**Figure 10:**
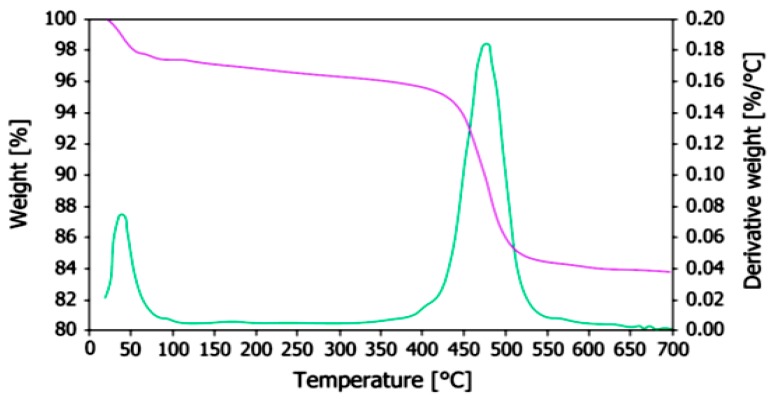
TGA curves of halloysite particles [84].

To measure weight changes in halloysite particles as a function of temperature, Thermal Gravimetric Analysis (TGA) was conducted. Results in Figure 10 indicate that the relative stability of halloysite reaches up to 400 °C. However, when the temperature surpasses the 500 °C mark, a weight loss of 15% occurs, which is attributed to the dehydration process due to the removal of interlayer water [73,83,84].

**Figure 11:**
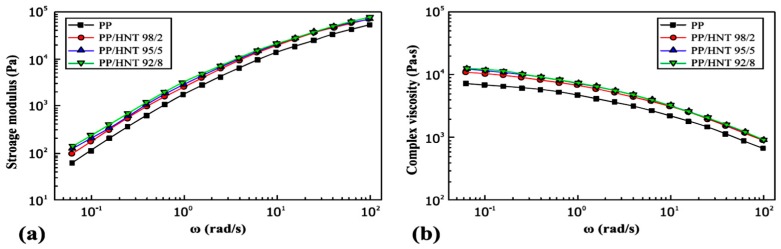
(**a**) Storage modulus (*G'*) and (**b**) complex viscosity (η*) as a function of frequency (ω) for pure PP and as-extruded PP/HNTs nanocomposites with different weight ratios [85].

Figure 11a clearly shows that the PP/HNTs nanocomposites show a higher storage modulus (*G'*) than pure PP over the entire frequency range. Furthermore, at high frequencies, the *G'* is unconstrained by the HNTs content in the PP/HNTs nanocomposites. By contrast, *G'* increases when HNTs content is increased to 5 wt % at low frequencies. However, no further increase in *G'* resulted when HNTs content increased to 8 wt %. Likewise, Figure 11b, shows higher complex viscosity (η*) for the PP/HNTs-based nanocomposites compared with the neat PP over the entire frequency range. Furthermore, the contents of HNTs exert a similar influence on the η* as in the *G'* above [85]. DMA analysis of HNTs/beeswax was examined, and the results showed a slight loss of beeswax crystallinity due to heating. The amount of HNTs entrapped in the pores and the shrinkage volume of wood samples were determined. The HNTs/beeswax mixtures can be considered a promising consolidant material for archaeological wood [86,87].

#### 6.4.2. HNTs and Poly(hydroxybutyrate-co-hydroxyvalerate)-Based Nanocomposites

**Figure 12:**
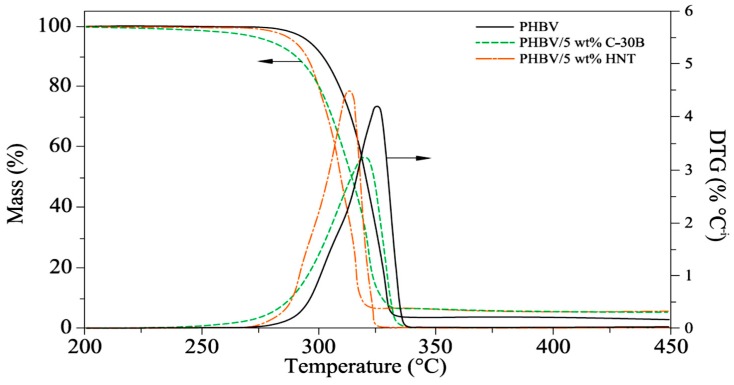
TGA and DTG curves of PHBV nanocomposites with 5 wt % nanoparticles [74].

Typical thermal gravimetric analysis TGA and derivative thermal gravimetric analysis DTG curves for PHBV/C-30B and PHBV/HNTs nanocomposites doped with 5 wt % nanoparticles are presented in Figure 12. According to the random chain scission reaction, the thermal degradation consisted of one weight loss step. As indicated, the thermal degradation is due to only single weight loss, which occurs between 290 °C and 340 °C for the PHBV nanocomposites and is clearly displayed in the TGA curves [74].

## 7. Technological Processes for Fabrication of HNTs-Polymer Nanocomposites and the Enhanced Properties

The versatility of HNTs for producing varieties of enhanced nanocomposites integrated with excellent processability and the exploitation of traditional and common technological processes for the production of HNTs-polymer nanocomposites and the enhanced properties consolidate its novelty. Table 5 shows the technological processes used in the manufacture of HNT-based nanocomposites with the range of HNT loading and the intended/achieved target properties for each composition.

For medical applications, it has been shown that even adding of 1 wt % HNTs to the cell culture did not result in cells’ death. Finally, the mechanism of cellular uptake was visualized and revealed that the nanoparticles concentrated around the cell nucleus after penetrating through the cell wall [52].

## 8. Conclusions

HNTs acted as a reinforcing nanofiller, resulting in a closely-compacted microstructure with an effectively better interaction between the HNTs and the host polymer by bringing a remarkable impact on the mechanical and thermal characteristics of polymers. Due to a lower price and availability, HNTs are a highly prospective material for upscaling composite productions of clay-based polymers. SEM micrographs affirm the possibility of achieving a uniform dispersal of HNTs in the matrix of polymers. However, in some cases, aggregation of HNTs was observed. Moreover, halloysite nanotubes exhibit enhanced strength compared with neat polymer properties. The tensile strength of the polymer increases directly with the increase in the content of HNTs up to 7 wt %, then declines. Moreover, the tensile strength increases with the inclusion of HNTs under a high strain loading rate. The modulus of rupture, also known as the ‘flexural strength’ as one of the measures for the tensile strength of polymeric materials, was found to increase with the HNTs’ inclusion compared with the pure polymer. Furthermore, the concentration of HNTs with a higher aspect ratio has an apparent impact on the flexural strength. However, HNTs as a nanofiller achieve a considerably better enhancement in flexure strength compared with other nanofillers. These features make HNTs a highly prospective material for upscaling composite productions of clay-based polymers. The above finding shows that the strengthening effect at low filler fractions is possible as a result of the build-up of an efficient organization of the crystalline stage in the halloysite. In particular, the modulus as a mechanical feature in addition to the hardness was substantially improved by the incorporation of HNTs. Briefly, HNTs can be considered as a very promising additive, and based on previous research, HNTs have shown tremendous improvements; and hopefully, more of these improvements will evolve.

## Figures and Tables

**Table 1:** Summary of the main properties [51,52].

No.	Property	Advantageous Features	Applications
1	Natural, nontoxic	Maintaining uniformly controlled release rate combined with initial over dosage prevention	Controlled and sustained release medicine
2	Fine particle size and superb dispersion	Realizable regardless of their forms such as; powders, creams, gels, or lotions, finally sprays form	Providing natural protection environment within the internal cavity ‘lumens’ of the nanotubes for the active agent during inharmonious and harsh material processing
3	High cation exchange capacity	Regeneration ability and increased efficacy	-
4	High aspect ratio	Trigger-capable release mode with adjustable release rate	Inhibiter, controlled medicine delivery
5	High porosity	Capable of loading multiple active agents simultaneously	Multiple active agents loading
6	High surface area	Reduces the volume of costly active agents	Pharmaceutical Industries
7	Non-swelling	Superior loading rates to other carriers, Fast adsorption rate and high adsorption capacity	Drug delivery, mechanical property enhancer
8	Biocompatible EPA 4A listed material	Biocompatibility: HNT has no cytotoxic effects which makes it suitable in drug delivery system. Tunable release: Incorporation HNT in drug delivery system enable them to sustainably release bioactive agents for various duration ranging from ten hours to months	1. Medical implants devices 2. Skin care products 3. Prolonged treatment drugs
9	Processability: Markedly impressive in comparison to other nanoclays	Easily dispersible: The surface of HNT is lightly loaded with hydroxyl groups that limit its capability of developing hydrogen bonding between particles ‘intra-particles’. This can help meet the full range of processing requirements by the appropriate industry. In contrast to the surface of platy clay that are heavily stacked with hydroxyls group	1. Can be used for manufacturing of interior and exterior parts 2. Enables the production of larger and/or thinner finished parts
10	Compatibility: Polymers oriented	The surface interaction between nanoclays and the following polymers is; powerful and robust: Polar biopolymers: like; polyacrylates and polyelectrolytes. Medium polarity polymers: like polyvinylchloride.	Polymers with enhanced mechanical properties, thermal stability and fire retardant composites
11	Controllability of release (Sustainable)	The internal cavity of the HNT called ‘lumen’ has the capability to store molecules and controllably releasing them. HNT mechanical and chemical stability is considerably higher compared to other nanoparticles classified as conventional, like alumina and porous silica	1. Smart materials (Polymeric composites with self-healing capability) 2. Regenerative medicine 3. Drug delivery system 4. Polymeric biocides materials (antimicrobial)

**Table 2:** Halloysite-polymer composites′ preparing methods [52].

No.	Name of the Method	Method Outline to Obtain the Composites	Reaction/Notes/Results in Such Composites
1	In situ polymerization	Dispersing fine halloysite tubes in a monomer	Monomers interact with the halloysite surface and form a uniform suspension
2	Solution casting	1. Halloysite dispersal in the polymer solution 2. Solvent evaporation	Sandwiched multilayer structures Forming of halloysite-rich layers Polymer polarity-based results
3	Direct addition/extruder blending	1. Addition of halloysite directly into the melted polymer 2. Blending either by (mechanical mixer and extruder)	Target: to obtain uniform distribution of the nanotubes in a polymer matrix
4	Deposition by layer	Layer-by-layer deposition in rotation of halloysite, polycations	Sequential substrate dipping in clay and polycation solutions was adopted to make the coating

**Table 3:** Effects of HEDA, silane and urea modifications on the mechanical characteristics of the HNTs/PP nanocomposites [82].

Blend Composition	σ_b_ (MPa)	E (MPa)
Neat PP	36.3 ± 0.4	1340 ± 45
PP + 5 wt % untreated HNT	36.5 ± 0.4	1440 ± 84
PP + 5 wt % HNT modified HEDA	36.5 ± 0.3	1547 ± 35
PP + 5 wt % HNT modified silane	36.2 ± 0.6	1495 ± 65
PP + 5 wt % HNT modified urea	36.5 ± 0.8	1449 ± 30

**Table 4:** Influence of 5 wt % of halloysite on PP crystallinity [82].

Sample	*T*_m_	Crystallinity (%)
Neat PP	166.0	46.1
PP + 5 wt % untreated HNT	163.0	41.0
PP + 5 wt % untreated HNT + 1 wt % DBMI	164. 4	42.2
PP + 5 wt % untreated HNT + 2 wt % DBMI	159.9	40.3
PP + 5 wt % untreated HNT + 3 wt % DBMI	162.9	43.2
PP + 5 wt % HNT modified HEDA	162.7	43.5
PP + 5 wt % HNT modified HEDA + 1 wt % DBMI	159.7	37.3
PP + 5 wt % HNT modified silane	166.1	56.3
PP + 5 wt % HNT modified silane + 1 wt % DBMI	165.0	35.8
PP + 5 wt % HNT modified urea	170.0	22.3
PP + 5 wt % HNT modified urea + 1 wt % DBMI	170.0	25.5

**Table 5:** Halloysite nanotubes-polymer nanocomposites in technology.

Reference	Matrix	HNTs (wt %)	Process	Results of Nanocomposites		
				HNTs’ Modification	Property	±%
[82]	PP	5	Injection molding (internal)	Untreated HNTs	*T_m_*	−2
				HNTs modified DBMI		−1
				HNTs modified HEDA		−2
				HNTs modified Silane		+0.06
				HNTs modified urea		+2
				Untreated HNTs	*X_C_*	−11
				HNTs modified DBMI		−6
				HNTs modified HEDA		−6
				HNTs modified Silane		+22
				HNTs modified urea		−51
				Untreated HNTs	Tensile strength	+1
				HNTs modified HEDA		+1
				HNTs modified Silane		−2
				HNTs modified urea		+1
				Untreated HNTs	Young′s modulus	+7
				HNTs modified HEDA		+15
				HNTs modified Silane		+12
				HNTs modified urea		+8
[85]	PP	0–8	Injection molding (extruder)		*T_C_*	+6
					DSC (cooling)	+5
					DSC (melting)	+3
					*X_C_*	+23
					TGA (weight loss)	+1
[88]	PP	1–10 phr	Injection molding (extruder)		Tensile strength	+23
[9]	PP	0–30 phr	Injection molding (extruder)		TGA (weight loss)	+8
[20]	EPDM	0–100 phr	Compound mold	M459	Tensile strength	+809
				M63	Elongation at break	+306
[21]	EPDM	0–100 phr	Compound mold		Tensile strength	+874
					Elongation at break	+306
[13]	xSBR	0–30 phr	Compound mold (co-coagulated)		Tensile strength	+53
					Elongation at break	−33
					Shore hardness	+45
[33]	EP	0–10	Casting		*T_g_*	+11
					Tensile strength	−3
[84]	EP	0–10			*T_g_*	−1
					Tensile strength	+8
[72]	PA6	0–6	Injection molding (extruder)		DSC (cooling)	+14
					*T_C_*	+13
					*X_C_*	+22
					*T_g_*	+9
					Tensile strength	+30
[19]	PA6	0–10 phr	Injection molding (extruder)		*X_C_* (Cooling at 40 °C/min)	+48
[59]	PA6	0–30	Compound molding (extruder)		*T_g_*	+2
					*T_C_*	−1
					*X_C_*	+8
[69]	PA12	0-10	Compression molding		TGA (weight loss)	+2
[8]	PS	0–5	Injection molding		DSC	−7
					TGA (weight loss)	+7
[57]	Starch	0-8	Injection molding (extruder)	M29	Tensile strength	+29
[31]	PSt	0–9	Casting	PEG	Tensile strength	+59
[73]	PHBV	0–5	Injection molding (extruder)		*X_C_*	+11
					*T_C_*	+2
					Tensile strength	+3
					*T_m_*	+2
					*X_C_*	+11
[79]	PBS	0–7			Tensile strength	+7

## Nomenclature

TGA	Thermogravimetric Analysis
DSC	Differential Scanning Calorimeter
DMA	Dynamic Mechanical Analysis
TEM	Transmission Electron Microscope
SEM	Scanning Electron Microscope
FTIR	Fourier Transform Infrared Spectroscopy
PP	Polypropylene
xSBR	Carboxylated Butadiene-Styrene Rubber
PA6	Polyamide 6
EPDM	Ethylene Propylene Diene Monomer
PS	Polystyrene
PSt	Potato Starch
EP	Epoxy
PA12	Polyamide 12
PHBV	Poly(hydroxybutyrate-co-hydroxyvalerate)
PBS	Poly(Butylene Succinate)
PVA	Polyvinyl Alcohol
T_p_	Peak temperature
T_m_	Melting temperature
T_c_	Crystallization temperature
T_g_	Glass transition temperature
σ_b_	Tensile strength
E	Young modulus
E'	Storage modulus
G'	Storage modulus
η*	Viscosity
HDTMA	Hexadecyltrimethylammonium bromide
HEDA	Hexadecyl-tri-methyl-ammonium-bromide
DBMI	4,4′-diphenylmethylene dimaleinimide
M100	Modulus at 100% elongation
TS	Tensile strength
EB	Elongation at break
E′	Storage modulus
tan δ	Loss tangent
X_C_	Crystallinity
